# Supplementary material for: Robot-Assisted versus Laparoscopic Gastrointestinal Surgery: A Systematic Review and Metanalysis of Intra- and Post-Operative Complications
Source: J Pers Med. 2023 Aug 25;13(9):1297. doi: 10.3390/jpm13091297 (PMC10532788; doi:10.3390/jpm13091297)
Supplement: Supplementary file 1 [file jpm-13-01297-s001.zip › jpm-2554928-supplementary.pdf]

Table S1. Characteristics of the included studies

Author	Year	Surgery	Robot	Inclusion criteria	Exclusion criteria	Outcomes	Results
**Baik et al. [29]**	**2008**	Rectal surgery	Da Vinci®	Rectal cancer	Clinical stage T4 or M1, significant lateral pelvic nodes, tumour infiltration into the anal sphincter complex, and no consent to randomization.	Skin-to-skin operative time, haemoglobin change between preoperative (1 to 2 weeks before the operation) and postoperative (1 day after the operation) times, days to first passing of flatus, length of hospital stay, complications, and conversion, intraluminal bleeding, intra-abdominal bleeding, back pain, scrotal swelling.	Two open conversions occurred in the laparoscopy group, mean operating time, haemoglobin change, conversion rate and the mean time to first passing of flatus were not significantly different between the two groups; length of hospital stay was shorter for the robotic group; the overall complication rate was higher for the robotic group.

**Patriti et al. [30]**	**2009**	Rectal surgery	Da Vinci®	Rectal adenocarcinoma irrespective to the stage of disease	Conversion to laparotomy, a preferred open approach.	Operative time, blood loss, conversion rate, perioperative complications, and length of hospital stay, anastomotic leak, wound infection, haemorrhage, prolonged ileus, urinary retention, enteritis.	Mean operating time, estimated blood loss and 30-day mortality were not significantly different between the two groups. The conversion rate was higher (19%) in the laparoscopic group.

**Jayne D et al. [31]**	**2019**	Rectal surgery	Not specified	Age ≥18 years; rectal adenocarcinoma amenable to curative surgery by low anterior resection, high anterior resection or abdomin-perineal resection; suitable for robotic-assisted or laparoscopic rectal resection.	Locally advanced cancers not amenable to curative surgery or requiring end multivesicular resection, synchronous colorectal tumours, co-existent inflammatory bowel disease, malignancy within the past 5 years, or pregnancy.	Primary outcome: conversion to laparotomy<br>Secondary outcomes:<br>intra- and postoperative complication, pathological outcomes, quality of life, bladder and sexual dysfunction, and oncological outcomes, intraoperative complications: damage to an organ, haemorrhage, and surgical equipment failure.<br>Thirty-day postoperative complications: anastomotic leak, surgical site infections and urinary complications. Six-month postoperative complications (after 30 days): anastomotic leak.	There were not statistically significant differences between robotic surgery and laparoscopic surgery with respect to conversion rate,<br>circumferential resection margin positivity,<br>intraoperative complications,<br>postoperative complications within 30 days and<br>postoperative complications within 6 months.

**Tolstrup et al. [32]**	**2018**	Rectal surgery	Da Vinci®	Primary, recurrent and advanced rectal cancers informed written consent.	Conversion to open surgery	Postoperative pain,<br>intraoperative and post-operative analgesic consumption, complications<br>According to the Clavien-Dindo score*	There were no significant differences in morphine consumption, in post-operative NRS score, in operating time, in length of hospital stay or postoperative complications. The conversion rate and the opioid consumption in the laparoscopic group was significantly higher than in the robot-assisted one.

**Debakey et al. [33]**	**2018**	Rectal surgery	daVinciSi®	Histological diagnosis of adenocarcinoma of rectum, no anaesthesiologic contraindications to minimally invasive surgery, age ≤75 years, ASA ≤2.	Metastatic disease, malignant bowel obstruction, and unresectable cancer.	Median time to passage of first flatus, days of hospital stay, complications, anastomotic leakage, ileus, wound problems and others, rate of reoperation, rate of readmission, and 30-day mortality.<br>According to the Clavien-Dindo score*.	The operative time, median time to passage of first flatus was longer in the robotic group.<br>estimated blood loss was statistically significantly lower in the robotic group.<br>The median length of hospital stay, post-operative complications and anastomotic leakage were no significant statistical differences between the two groups.

**Kim MJ et al. [34]**	**2018**	Rectal surgery	Da Vinci®	Rectal adenocarcinoma located within 9 cm of the anal verge	Cancer invading adjacent organs (T4), distant metastasis (M1), severe concomitant disease that might limit compliance or completion of the protocol, any other malignancy, pregnant or breastfeeding females, hereditary colorectal cancer, and emergency operation.	Primary outcome: quality of total mesorectal excision specimen. Secondary outcomes: circumferential and distal resection margins, the number of harvested lymph nodes, morbidity, bowel function recovery, and quality of life, anastomotic leakage, ileus, acute voiding difficulty, stoma-related complication, wound discharge, bleeding and others.	Operative time and estimated blood loss were significantly higher in the robot-assisted group than in the laparoscopic group.<br>The quality of total mesorectal excision, time to first flatus passage, first defecation, resumption of diet, the mean admission duration and pain scores were similar between the two groups.

**Park JS et al. [25]**	**2012**	Colectomy	Da Vinci®	Newly diagnosed right-sided colonic carcinoma, acquired informed consent.	No consent to randomization, unfit for operative treatment, acute surgical emergency, distant metastasis on preoperative evaluation and advanced tumour with adjacent organ invasion requiring en-bloc multiple organ resection.	Primary outcome: length of hospitalization. Secondary outcomes: duration of operation, morbidity, postoperative pain, hospital costs and pathological quality of the specimen, wound infection, anastomotic leakage, intra-abdominal abscess, ileus and bleeding.	Conversion rate to open surgery, estimated blood loss, mean time to first passage of flatus, length of hospital stay, intraoperative transfusion, pain score and major complication rate were similar in both groups. Operative time and total costs were significantly higher in the robot-assisted group than in the laparoscopic one.
**Ojima et al. [40]**	**2021**	Gastric surgery	da Vinci Si or Xi Surgical System®.	Histological diagnosis of gastric carcinoma, resectable cancer, age between 20 and 90 years, Eastern Cooperative Oncology	Synchronous or metachronous malignant neoplasms other than carcinoma in situ, pregnant or breastfeeding, severe mental	Primary outcome: incidence of postoperative intra-abdominal infectious complications. Secondary outcomes: incidence of any complications, surgical results, postoperative courses, and oncologic outcomes.<br>According to the Clavien-Dindo score*.	Overall incidence of intra-abdominal infectious complications, conversion rate,<br>median time to ambulation, first drinking, first oral intake, and

				Group performance status of 0 or 1, body mass index <35, no history of gastrointestinal surgery that may affect protocol surgery, no history of chemotherapy or radiotherapy, normal function of the major organs and proven written informed consent.	illness, continuous systemic corticosteroid therapy, history of myocardial infarction or unstable angina pectoris within 6 months, uncontrollable hypertension, uncontrollable type 1 or 2 diabetes or administration of insulin, respiratory disease requiring continuous oxygen therapy, and history of deep-vein thrombosis.		postoperative stay were not significantly different between the two groups.<br>The median time to first flatus, the overall incidence of postoperative complications of grade II or higher and the number of postoperative analgesics were significantly lower in the robot-assisted group.

**Lu J et al. [9]**	**2021**	Gastric surgery	Da Vinci®	Aged 18 to 75 years, histologically proven gastric cancer, clinical stage cT1–4aN0/þM0.	History of previous neoadjuvant chemotherapy.	Primary outcome: 3-year disease-free survival rate. Short-term clinical outcomes: intraoperative outcomes, postoperative recovery course, morbidity, quality of lymphadenectomy, adjuvant chemotherapy completion status, and cost difference. Within 30 postoperative days: morbidity and mortality graded using the Clavien-Dindo classification system.<br><br>Surgical morbidity: abdominal bleeding, anastomotic leakage, ileus, gastroplegia, wound infection and peritoneal infection.<br><br>Medical morbidity: pneumonia, cardiovascular system, liver system, urinary system and deep vein thrombosis, unplanned readmission.	Total operative time was longer in the robot-assisted group than in the laparoscopic one.<br><br>The intraoperative blood loss, the postoperative recovery time, the time to ambulation, to first flatus, liquid intake, the overall postoperative morbidity rate, and incidence of pneumonia were significantly lower in the robot-assisted group.<br><br>There was no significant difference in the length of hospital stay, reoperation rate, incidence of surgical morbidity (incidence of abdominal

							haemorrhage, anastomotic leakage, ileus, gastroplegia, wound infection, and peritoneal infection) and incidence of medical morbidities (postoperative cardiovascular system, liver system, urinary system, and deep vein thrombosis complications) between the two groups.

**Dhanani et al. [26]**	**2021**	Hernia repair	Not specified	Adult patients undergoing elective minimally invasive ventral hernia repair with defect < 12 cm wide, able to tolerate pneumoperitoneum.	Patients unlikely to survive more than 2 years, to follow up, unable to speak either English or Spanish.	Complications, hernia occurrence, readmission, reoperation, functional status.<br>Wound complication: surgical site infection. Seroma, hematoma, wound dehiscence. Ileus, abdominal wall abscess.	There was no difference in wound complication rate and in functional status in patients undergoing robotic and laparoscopic surgery.
**Olavarria OA et al. [27]**	**2020**	Hernia repair	Not specified	Age >18 years, a ventral hernia defect less than 12 cm, likely toleration pneumoperitoneum, no history of open abdomen or extensive lysis of adhesions, no active infection such as mesh infection.	Severe comorbid conditions that limit survival beyond two years, patients unlikely to follow up, and non-English and non-Spanish speakers.	Primary outcome: number of days in hospital within 90 days after surgery. Secondary outcomes: emergency department visits, operating room time, wound complications, hernia recurrence, reoperation, quality of life, and costs, surgical site infections, surgical site occurrences and hernia recurrence.	The median number of days in hospital at 90 days after surgery, conversions rate, readmission rate, emergency department visits, complications and pain scores did not differ between groups.<br>Robot-assisted group had significantly longer mean operating room

							times than the laparoscopic one.
**Petro CC et al. [28]**	**2021**	Hernia repair	Da Vinci Si or Xi ®	Age ≥18 years, elective setting with primary or incisional midline ventral hernias of an anticipated width of 7 cm or less who were candidates for minimally invasive hernia repair.	Conversion to open surgery	Primary outcome: 30% difference in the NRS on the first postoperative day.<br>Secondary outcomes: Patient-Reported Outcomes Measurement Information System Pain Intensity short form, hernia-specific quality of life, operative time, wound morbidity, recurrence, length of stay, and cost.<br>Pulmonary embolism, readmission, reoperation and surgical site occurrence.	Robotic operations had a longer median operative time and higher costs than laparoscopic approach.<br>Preoperative or postoperative NRS score, postoperative Patient-Reported Outcomes Measurement Information System, hernia-specific quality-of-life scores, length of stay and complication rates, were similar in both groups.

**Morino M et al. [39]**	**2006**	Nissen fundoplication	DaVinci®	Clinical GORD that necessitated surgery according to the criteria of Hinder, ASA 1-2.	Giant hiatal hernia (>6 cm on preoperative barium meal), ASA 3-4, previous upper abdominal surgery, contraindications to pneumoperitoneum.	Intraoperative and postoperative complications, length of hospital stay, total operating time (including anaesthesia) and operative skin-to-skin time (included time needed for wrapping the arms and positioning the robot cart), cost evaluation (included the hospital stay, the use of the OR and the cost of surgical tools and excluded the initial cost of the robot).<br><br>Postoperative: mild transient dysphagia (at 1 month), endoscopic oesophagitis (at 6 months).	There were no intraoperative and postoperative complications in both laparoscopic and robotic groups. Total operating time and skin-to-skin time were significantly shorter for conventional laparoscopy. The cost of a robot-assisted procedure was significantly higher than that for standard laparoscopic fundoplication.
**Corrigan N et al. [35]**	**2018**	Rectal surgery (ROLARR)	Not specified	Age ≥ 18 years, able to provide written informed consent and to complete required questionnaires, diagnosis of rectal cancer	Benign lesions of the rectum, cancers of the anal canal, locally advanced cancers not amenable to curative surgery or requiring en bloc	Primary endpoint: intra-operative conversion to open surgery.	Robotic-assisted laparoscopic surgery does confer an advantage over standard laparoscopic surgery in terms of the risk of conversion to open surgery, when performed by an

				amenable to curative surgery by high anterior or low anterior or abdominoperineal resection and suitable for resection by either standard or robotic-assisted laparoscopic procedure, ASA ≤ 3.	multivisceral resection, synchronous colorectal tumors requiring multisegmented surgical resection, co-existent IBD, clinical or radiological evidence of metastatic spread, concurrent or previous diagnosis of invasive cancer within 5 years that could confuse diagnosis, history of psychiatric or addictive disorder, pregnancy, participation in another rectal cancer clinical trial relating to		operating surgeon with a substantial level of previous experience with robotic surgery, regardless of their level of previous experience in standard laparoscopic surgery.

					surgical technique.		
**Mäkelä-Kaikkonen J et al. 2016 [36]**	**2016**	Rectal surgery	DaVinci ®	Female, age ≥ 18 and ≤ 85 years, ASA 1-3, uncomplicated rectal prolapsed or symptomatic isolated rectal prolapsed, intussusceptions, enterocele (with obstructive defecation and/or faecal incontinence).	Male, ASA 4-5, previous major pelvic surgery, suspicion of frozen pelvis, pregnancy or future plans for pregnancy.	Secondary outcomes: duration of surgery, early complications, preparation time (from the patient arrival to the OR to the start of the operation including anaesthesia), operating time (the duration of surgery from the first cut to the last stitch), operating room time (the duration of the overall procedure, from arrival of the patient to the OR to the time of leaving it), perioperative bleeding, conversion rate, in-hospital stay, VAS during the hospital stay and at 2 weeks postoperatively.<br><br>Vascular complication: minor bleeding from the iliac vein; minor complications: fever, hematoma of the rectus abdominis, transient pain in the perineum.	There was no difference in the robotic-assisted and laparoscopic operating time, in robotic-assisted surgery there was a low complication rate with minor bleeding from the iliac vein (a complication which may be difficult to treat laparoscopically, but treated by robot-assisted suturing, without conversion to open surgery).

**Feng Q et al. [37]**	**2022**	Rectal surgery	DaVinci ®	Single middle or low rectal cancer (inferior tumour edge ≤ 10 cm from the anal verge), age ≥ 18 and ≤ 80 years, ASA 1-3, histologically proven rectal adenocarcinoma, tumour assessed as cT1-T3, N0-N1 or ycT1-T3 Nx after preoperative RT or CT, no evidence of distant metastasis, no other malignancies in medical history, suitable for both robotic and	Clinical complete response after preoperative RT or CT, tumour assessed as cT1N0 and suitable for local excision, emergency surgery, multiple colorectal tumours or synchronous colon surgery, hereditary colorectal cancer, coexisting IBD, pregnancy or lactation, receiving treatment other than preoperative RT or CT.	Secondary endpoint: intraoperative and 30-day postoperative complications, operating time, estimated blood loss, conversion rate, 30-day postoperative mortality.<br>Disease-free survival and overall survival, time to first flatus, postoperative hospital stay (included the time of readmission within 30 days after surgery for the primary tumour), total costs of hospitalisation (from admission to discharge, also readmission and reoperation within 30 days post-surgery, but not for ileostomy closure) and direct costs of surgery (expenses during surgery) and postoperative costs (after surgery for the primary tumour before discharge).<br>30-day postoperative complications evaluated according to the Clavien-Dindo* classification, included anastomotic leakage. Intraoperative: significant bleeding, damage to organs or structures, anastomotic complications, iatrogenic perforation.	Intra and postoperative complications of Clavien-Dindo were less frequent in the robotic group than in the laparoscopic one within 30 days after surgery, also anastomotic leakage. Patients after robotic surgery had a faster time to first flatus and a shorter postoperative hospital stay, a minor conversion rate and estimated blood loss, higher total costs of hospitalisation and direct costs of surgery but lower postoperative costs. No difference in preoperative costs

				laparoscopic surgery.			and operating time between robotic and laparoscopic groups.
**Draaisma et al. [38]**	**2006**	Nissen fundoplication	DaVinci®	Age > 18 years, presence of GORD (diagnosed with medical history, barium esophagram series, upper endoscopy, esophageal manometry and 24h pH monitoring).	General contraindication for laparoscopy or previous abdominal surgery, psychiatric diseases.	Operative time (from first skin incision to the final closure of the last skin incision), early/ intraoperative and late (more than 30 days) complications, estimated blood loss, length of fundoplication, reoperations, hospital stay, conversion rate.<br>Intraoperative: small liver capsule tears, small spleen capsule tears, pneumothorax; postoperative: pneumonia, urinary tract infection.	There were no differences in intra and postoperative complications and hospital stay. In robotic surgery, operating times were longer than in standard laparoscopic procedures and costs were higher. Robotic use was not supported in Nissen fundoplication because results appear to be similar, but operations take longer and more expensive.

**Kudsi OY et al. [41]**	**2016**	Cholecystectomy	Da Vinci®	Age ≥ 18 and ≤ 80 years, diagnosis of symptomatic gallbladder disease, will-ingness to participate in the study and provide informed consent.	Requirement of emergency procedure, acute cholecystitis, pregnancy, presence of upper midline visible abdominal scars or keloid, umbilical hernia or prior umbilical hernia repair, intolerance to Trendelenburg position or pneumoperito neum, cirrhosis, mental impairment that would preclude giving informed consent.	Operative time, estimated blood loss, length of hospital stay, conversion rate, intraoperative complications, postoperative complications and readmission and re-operation through 3 months, quality of life<br><br>Postoperative: bile leakage, wound infection, IBD, and deep vein thrombosis/pulmonary embolism.	In both groups there weren't conversion to open surgery and the perioperative outcomes were similar. In robotic surgery there was an increased operative time and more satisfaction in patients.

*Clavien-Dindo score (reoperation for bleeding or leak, explorative laparotomy or diagnostic laparoscopy, intra-abdominal abscess, sepsis, pleural exudate with drainage tube, kidney failure with dialysis, pneumonia, pulmonary failure, deep vein thrombosis, pulmonary embolism, urinary tract infection, arrhythmia, complications with stoma, superficial wound infection/dehiscence, and postoperative gastrointestinal paralysis). NRS (Numeric Rating Scale). GORD (Gastro-Oesophageal Reflux Disease). IBD (Inflammatory Bowel disease).

Figure S1. Sensitivity analysis of intra and postoperative complications.

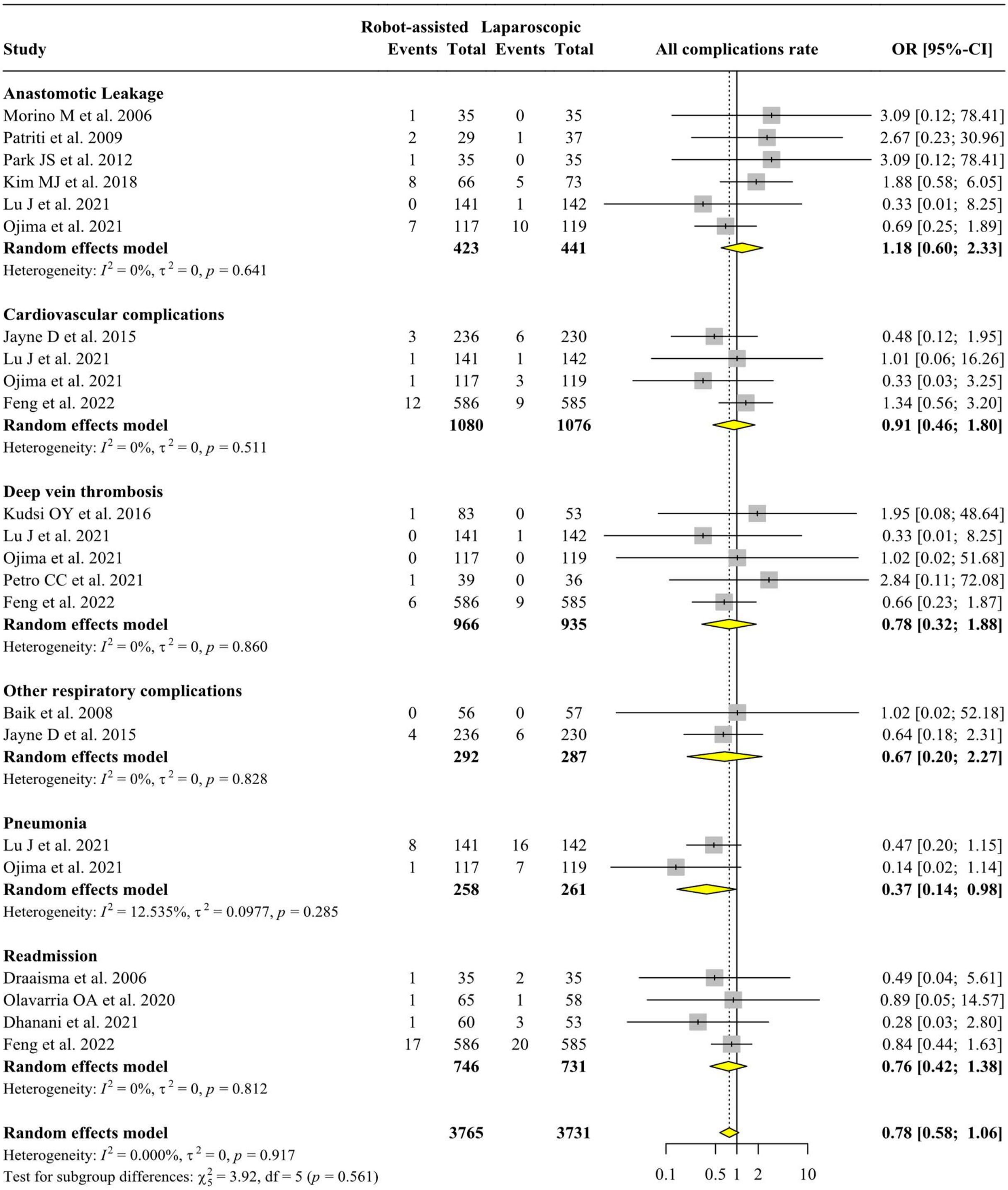

From the top: anastomotic leakage rate [9,25,30,34,39,40]; cardiovascular complications rate [9,31,37,40]; deep vein thrombosis rate [9,28,37,40,41]; other respiratory complications rate [29,31]; pneumonia rate [9-40]; readmission rate [26,37,38,40]; overall analysis [9,25,26,28-31,34,37-41].

Figure S2. Subgroup analysis of postoperative complications.

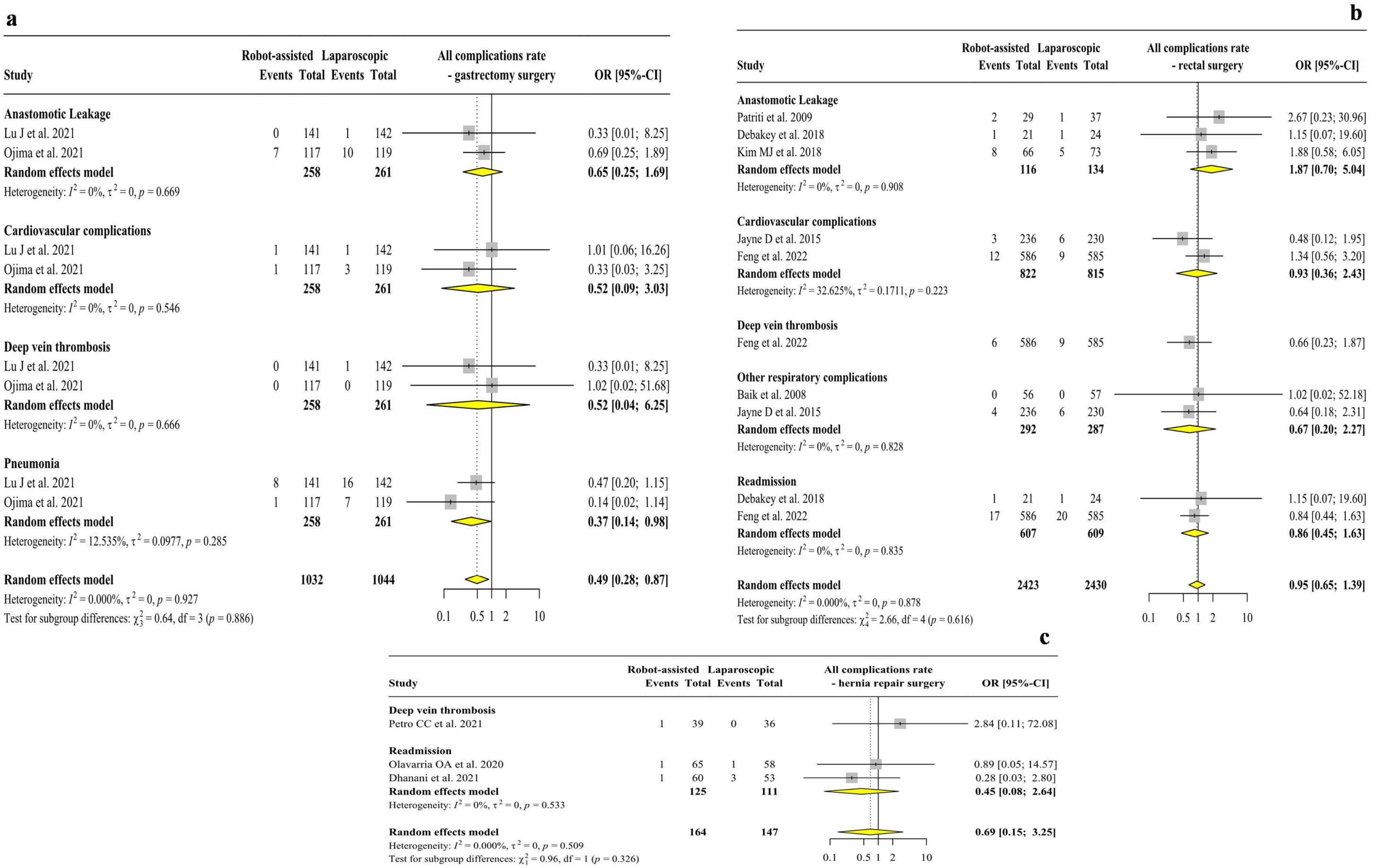

a) complications rate in gastrectomy surgery, from the top: anastomotic leakage [9,40], cardiovascular complications [9,40], deep vein thrombosis [9,40], pneumonia [9,40]; overall [9,40]; b) complications rate in rectal surgery, from upper to lower: anastomotic leakage [30,33,34], cardiovascular complications [31,37], deep vein thrombosis, other respiratory complications [29,31], pneumonia [33,37]; overall [29,31,33,37]; c) complications rate in hernia repair surgery, from upper to lower: deep vein thrombosis, pneumonia [26,27], overall [26-28].

Figure S3. Sensitivity analysis of all outcomes.

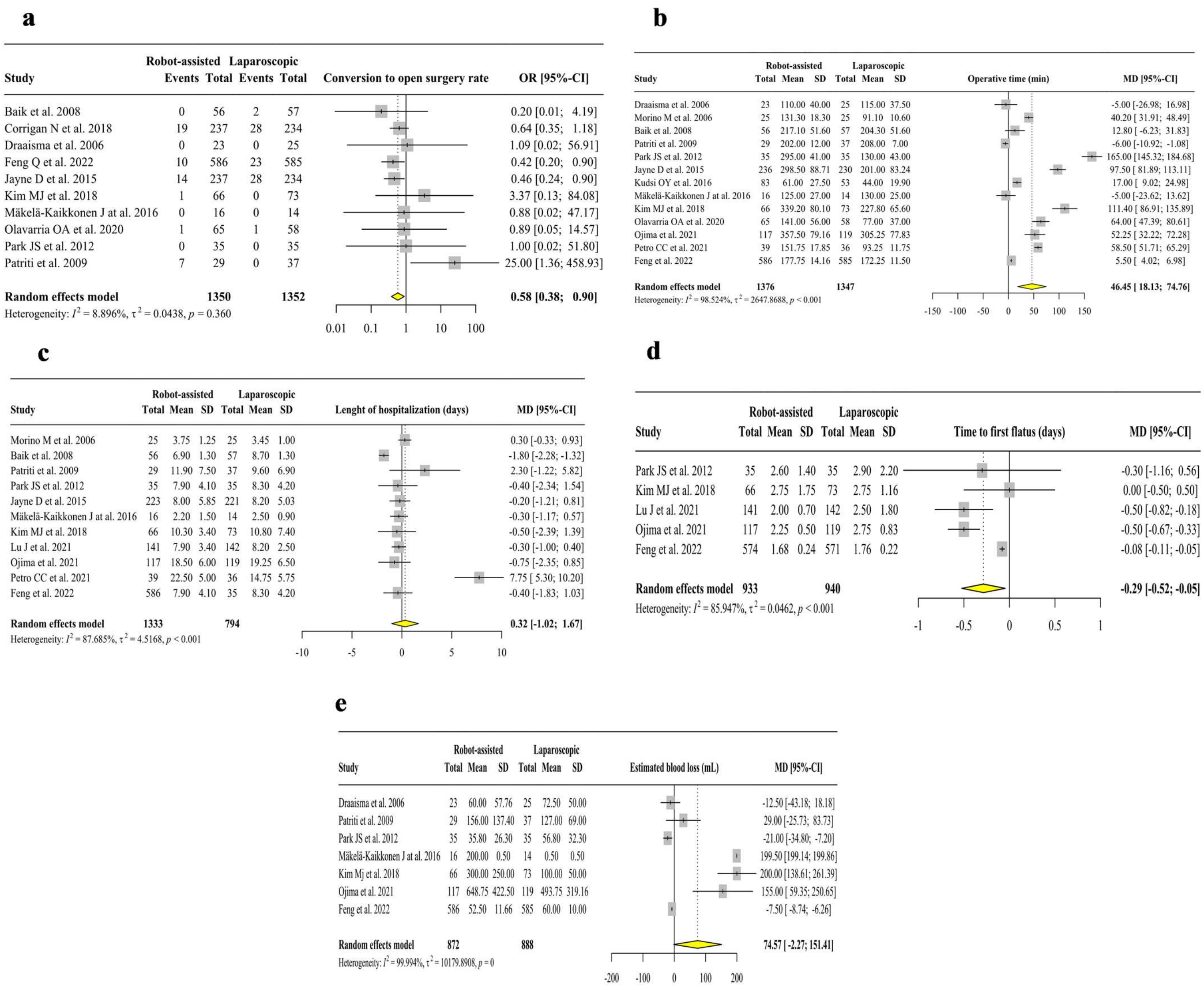

a) analysis of conversion to open surgery rate [25,27,29,30,31,34-38]; b) analysis of operative time (in minutes) [25,27-31,34,36-41]; c) analysis of length of hospitalization (in days) [9,25,28-31,34,36,37,39,40]; d) analysis of time to first flatus [9,25,34,37,40]; e) analysis of estimated blood loss (in mL) [25,30,34,36-38,40].

Figure S4. Subgroup analysis of conversion to open rate surgery, operative time, length of hospitalization, time to first flatus, and estimated blood loss.

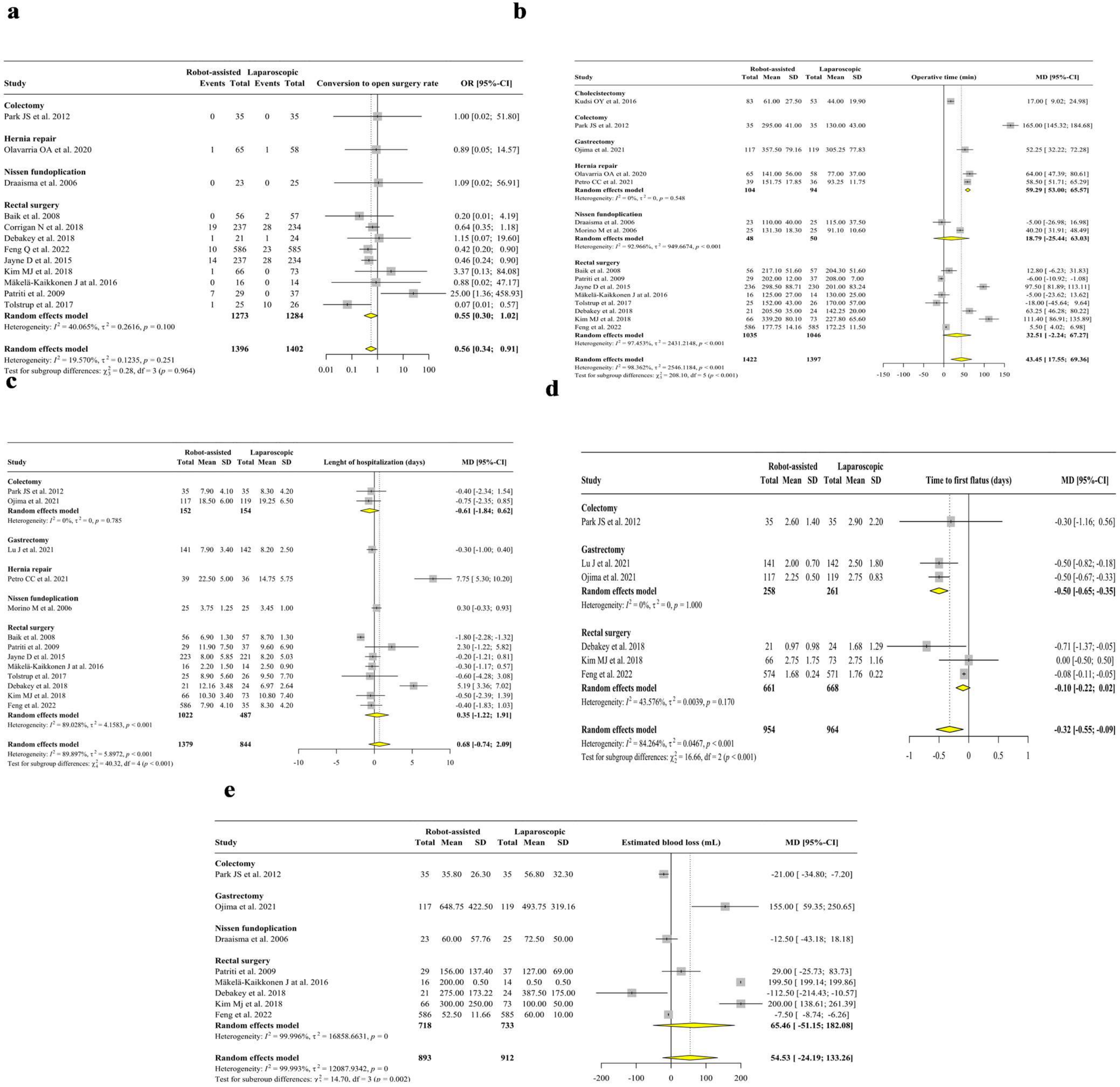

a) analysis of conversion to open surgery rate, from the top: colectomy [25], hernia repair [27], Nissen's fundoplication [38], rectal surgery [29-37], overall [25,27,29-38]; b) analysis of operative time (in minutes), from the top: cholecistectomy [41], colectomy [25], gastrectomy [40], hernia repair [27,28], Nissen fundoplication [38,39], rectal surgery [29-34,36,37], overall [25,27-34,36-41] ; c) analysis of length of hospitalization (in days), from the top: colectomy [25,40], gastrectomy [9], hernia repair [28], Nissen fundoplication [39], rectal surgery [29-34,36,37], overall [9,25,29-34,36,37,39] ; d) analysis of time to first flatus (in days), from the top: colectomy [25], gastrectomy [9, 40], rectal surgery [33,34,37], overall [9,25,33,34,37,40] ; e) analysis of estimated blood loss (in mL), from the top: colectomy [25], gastrectomy [40], Nissen fundoplication [38], rectal surgery [30,33,34,36,37], overall [25,40,30,33,34,36-38].**Figure S5**. Trial sequential analysis of each outcome.

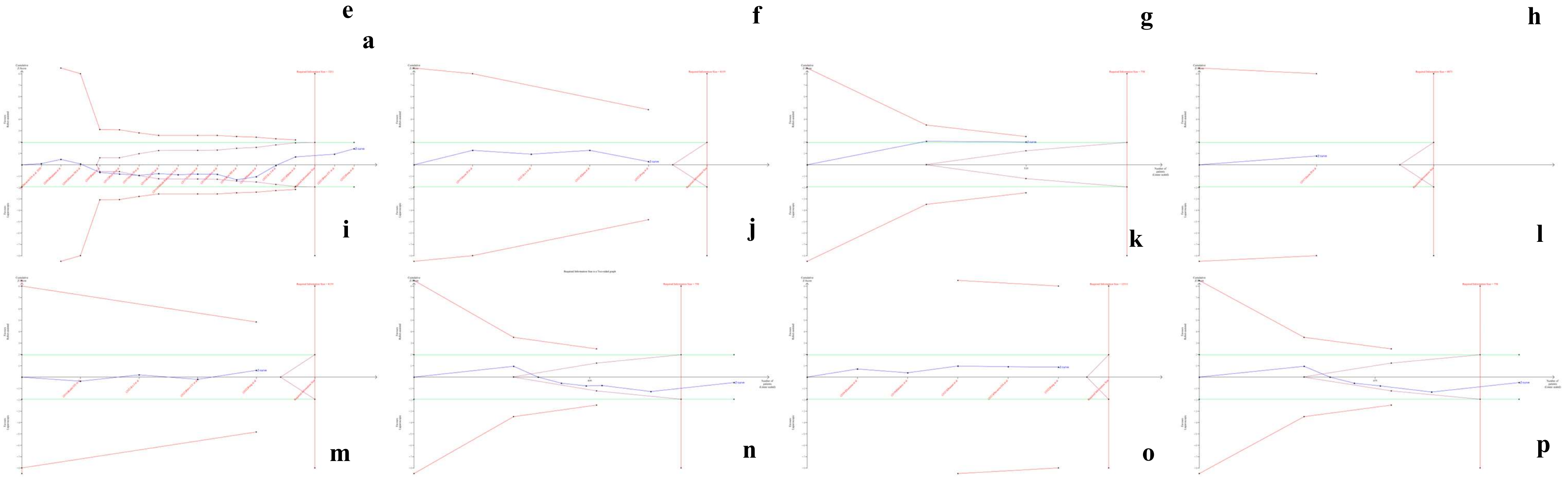

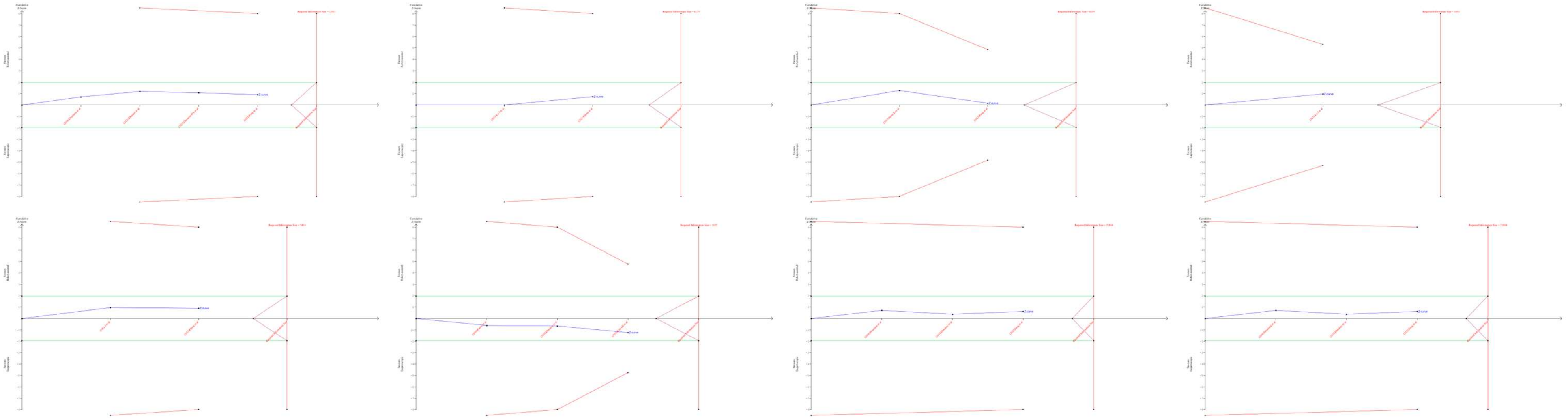

a) TSA of overall complications; b) TSA of cardiovascular complications; c) TSA of pneumonia; d) TSA of other respiratory complications; e) TSA of deep vein thrombosis; f) TSA of anastomotic leakage; g) TSA of readmission rate; h) TSA analysis of sensitivity analysis of anastomotic leakage; i) TSA analysis of sensitivity analysis of readmission rate; j) TSA of subgroup analysis of cardiovascular complications in gastrectomy procedures; k) TSA of subgroup analysis of cardiovascular complications in rectal surgery; l) TSA of subgroup analysis of deep vein thrombosis rate in gastrectomy procedures; m) TSA of subgroup analysis of anastomotic leakage in gastrectomy procedures; n) TSA of subgroup analysis anastomotic leakage in rectal surgery; o) TSA of subgroup analysis of readmission rate in rectal surgery; p) TSA of subgroup analysis of readmission rate in hernia repair procedures.

Figure S6. Trial sequential analysis of conversion to open surgery rate, operative time, length of hospitalization, and time to first flatus.

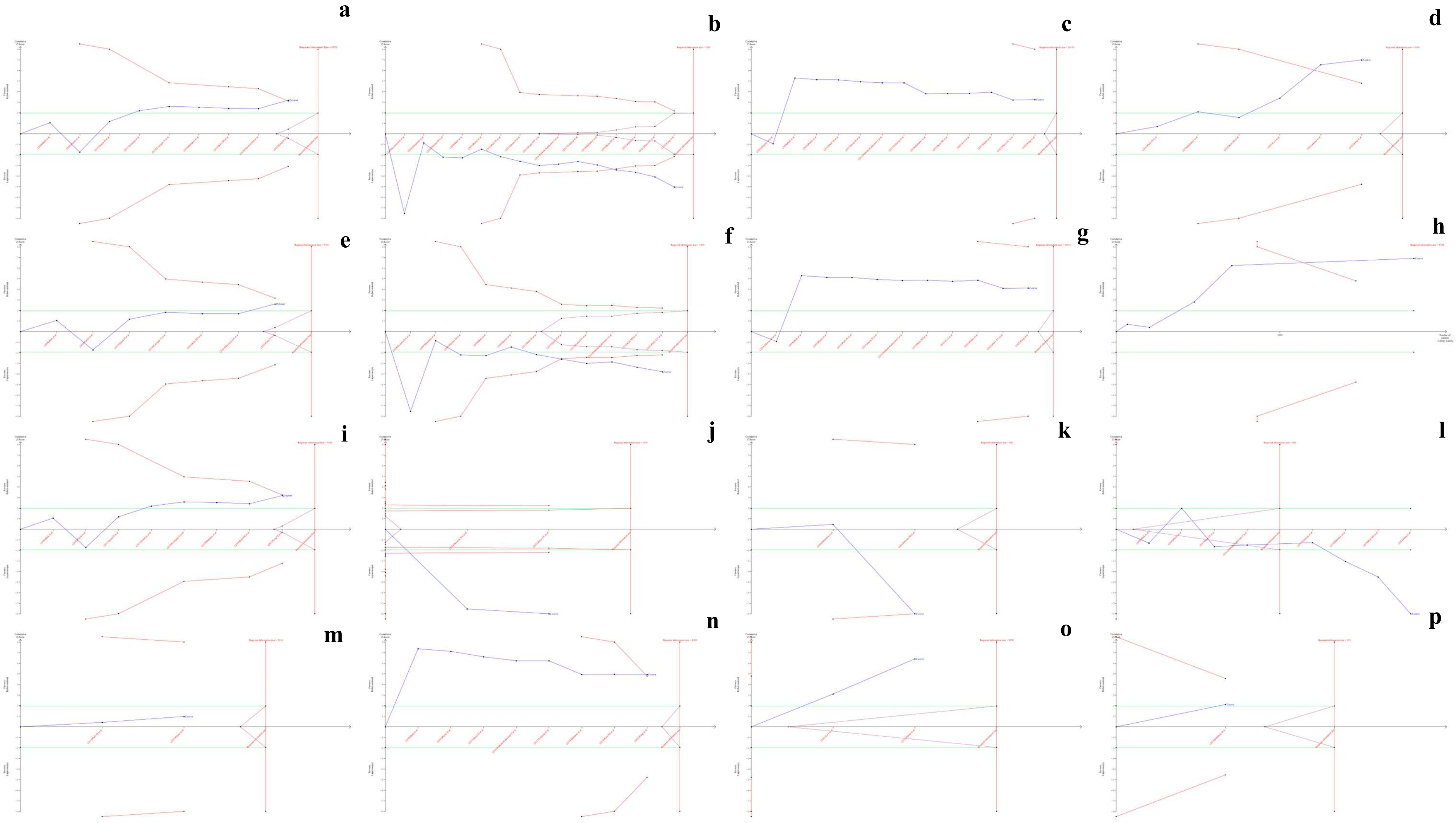

a) TSA of conversion to open surgery rate; b) TSA of operative time; c) TSA of length of hospitalization; d) TSA of time to first flatus; e) TSA of sensitivity analysis of conversion to open surgery rate; f) TSA of sensitivity analysis of operative time; g) TSA of sensitivity analysis of length of hospitalization; h) TSA of sensitivity analysis of time to first flatus; i) TSA of conversion to open surgery rate in rectal surgery; j) TSA of operative time in hernia repair surgery; k) TSA of operative time in Nissen's fundoplication surgery; l) TSA of operative time in rectal surgery; m) TSA of length of hospitalization in colectomy procedure; n) TSA of length of hospitalization in rectal surgery; o) TSA of time to first flatus in gastrectomy surgery; p) TSA of time to first flatus in rectal surgery.

Figure S7. Trial sequential analysis (TSA) of estimated blood loss, quality of life, mortality rate, pain after one day and one month.

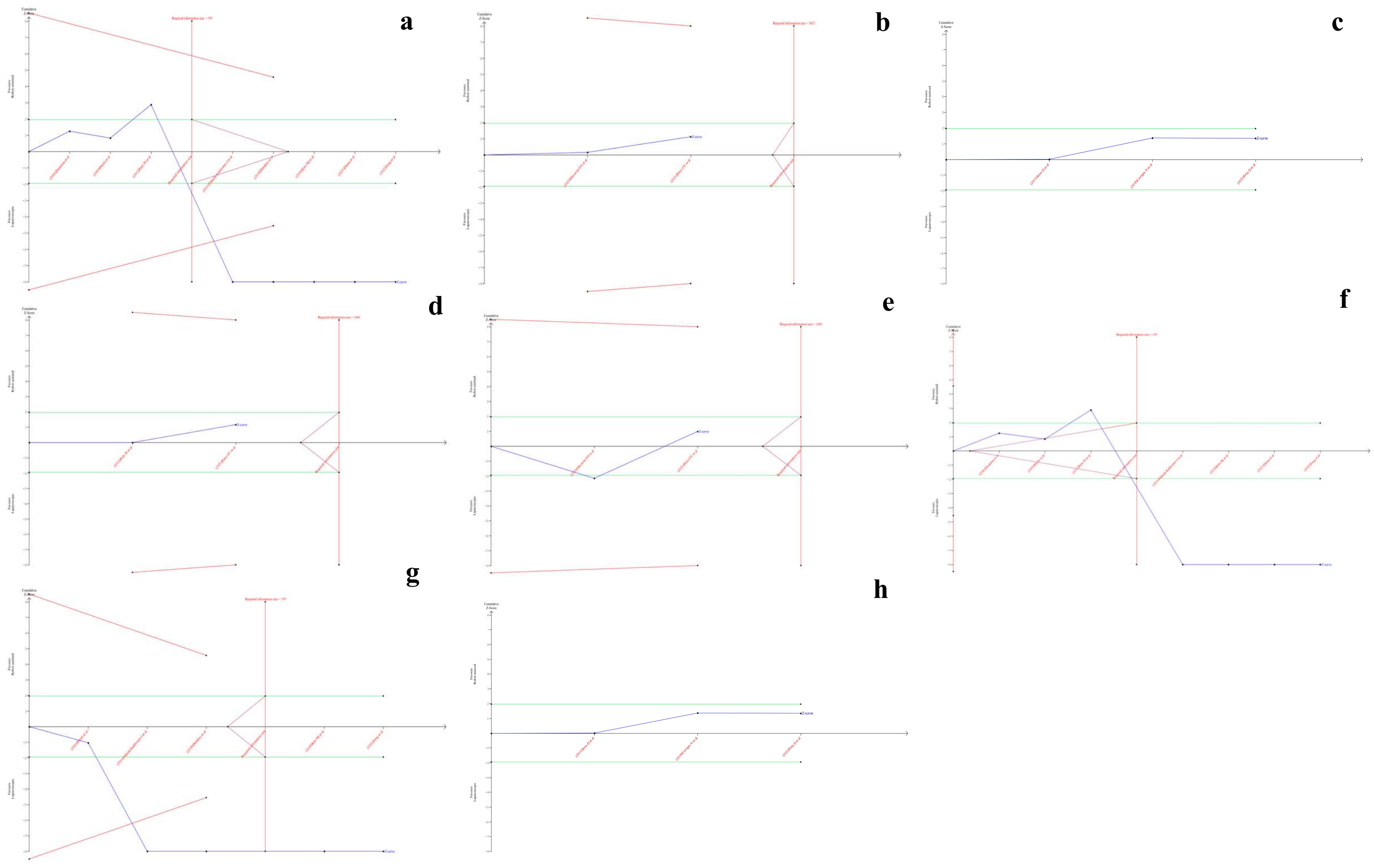

a) TSA of estimated blood loss; b) TSA of quality of life; c) TSA of mortality rate; d) TSA of pain at one day after surgery; e) TSA of pain at one month after surgery; f) TSA of sensitivity analysis of estimated blood loss; g) TSA of estimated blood loss in rectal surgery; h) TSA of mortality rate in rectal surgery.

Table S2. Fragility indexes of dichotomous outcomes for each study.

Authors	Conversion to open surgery rate	Cardiovascular complications	Deep vein thrombosis	Other respiratory complications	Pneumonia	Anastomotic Leakege	Readmission	Mortality	Median (Min-Max)
Baik et al. 2008 [29]	6	-	-	8	-	-	-	8	8 (6-8)
Corrigan N et al. 2018 [35]	4	-	-	-	-	-	-	-	4 (4-4)
Debakey et al. 2018 [33]	5	-	-	-	-	5	5		5 (5-5)
Dhanani et al. 2021 [26]		-	-	-	-	-	4		4 (4-4)
Draaisma et al. 2006 [38]	6	-	-	-	-	-	6		6 (6-6)
Feng Q et al. 2022 [37]	2	6	5	-	-	-	8	7	6 (2-8)
Jayne D et al. 2015 [31]	2	4	-	5	-	-	-	7	4 (2-7)
Kim MJ et al. 2018 [34]	6	-	-	-	-	3	-		3 (3-6)
Kudsi OY et al. 2016 [41]	-	-	6	-	-	-	-	-	6 (6-6)
Lu J et al. 2021 [9]	-	7	7	-	2		-	-	4.5 (1-7)
Mäkelä-Kaikkonen J at al. 2016 [36]	6	-	-	-	-	-	-		5.5 (5-6)
Morino M et al. 2006 [39]	-	-	-	-	-	6	-	-	6 (6-6)
Ojima et al. 2021 [40]	-	5	8	-	1	5	-	8	6.5 (1-23)
Olavarria OA et al. 2020 [27]	6	-	-	-		-	6		6 (6-6)
Park JS et al. 2012 [25]	7	-	-	-	-	6	-	7	6.5 (6-7)
Patriti et al. 2009 [30]	2	-	-	-	-	4	-	6	4.5 (2-6)
Petro CC et al. 2021 [28]	-	-	7	-	-	-	-	-	7 (7-7)
Tolstrup et al. 2017 [32]	3	-	-	-	-	-	-	-	5 (3-7)
Median (Min-Max)	5.5 (2-7)	5.5 (4-7)	6 (5-8)	6.5 (5-8)	1.5 (1-2)	5 (3-7)	6 (4-8)	7 (6-8)	

Min minimum; Max maximum
